# PCSK9 Levels and Metabolic Profiles in Elderly Subjects with Different Glucose Tolerance under Statin Therapy

**DOI:** 10.3390/jcm10050994

**Published:** 2021-03-02

**Authors:** Kari A. Mäkelä, Jari Jokelainen, Ville Stenbäck, Juha Auvinen, Marjo-Riitta Järvelin, Mikko Tulppo, Juhani Leppäluoto, Sirkka Keinänen-Kiukaanniemi, Karl-Heinz Herzig

**Affiliations:** 1 Research Unit of Biomedicine, Medical Research Center, Faculty of Medicine, University of Oulu and Oulu University Hospital, 90014 Oulu, Finland; ville.stenback@oulu.fi (V.S.); mikko.tulppo@oulu.fi (M.T.); juhani.leppaluoto@oulu.fi (J.L.); 2 Center for Life Course Health Research and Medical Research Center, Faculty of Medicine, University of Oulu and Oulu University Hospital, 90014 Oulu, Finland; jari.jokelainen@oulu.fi (J.J.); juha.auvinen@oulu.fi (J.A.); m.jarvelin@imperial.ac.uk (M.-R.J.); sirkka.keinanen-kiukaanniemi@oulu.fi (S.K.-K.); 3 Biocenter Oulu, University of Oulu, 90014 Oulu, Finland; 4 MRC Centre for Environment and Health, Department of Epidemiology and Biostatistics, School of Public Health, St Mary’s Campus, Norfolk Place, London W2 1PG, UK; 5 Unit of Primary Care, Oulu University Hospital, 90029 Oulu, Finland; 6 Institute of Pediatrics, Poznan University of Medical Sciences, 60-512 Poznan, Poland

**Keywords:** PCSK9, prediabetes, type 2 diabetes, LDL cholesterol, statin therapy, elderly, cohort study

## Abstract

Proprotein convertase subtilisin/kexin type 9 (PCSK9) degrades low-density lipoprotein cholesterol (LDL-C) receptors, and thus regulates the LDL-C levels in the circulation. Type 2 diabetics often have elevated LDL-C levels. However, the functions of PCSK9 in patients with alterations of glu-cose metabolism and statin therapy are still unclear. Method: we investigated a large cohort of 608 subjects, born in 1945 in Oulu, Finland (Oulu Cohort 1945). We studied the effects of PSCK9 lev-els with different glucose tolerances (normal glucose tolerance (NGT), prediabetes (PreDM) or type 2 diabetes (T2D)) with and without statin medication, and analyzed clinical data, NMR metabolomics and PCSK9 plasma levels. Results: PCSK9 plasma levels did not significantly differ between the three groups. Statin therapy significantly increased the PCSK9 levels in NGT, PreDM and T2D groups compared with subjects with no statins. In the NGT group, negative associations between PCSK9 and LDL-C, intermediate-density lipoprotein cholesterol (IDL-C), very low-density lipoprotein cholesterol (VLDL-C), total cholesterol and LDL and IDL triglycerides were observed under statin medication. In contrast, in the PreDM and T2D groups, these associa-tions were lost. Conclusions: our data suggest that in subjects with abnormal glucose metabolism and statin therapy, the significant PCSK9-mediated effects on the lipid metabolites are lost com-pared to NGT subjects, but statins reduced the LDL-C and VLDL-C levels.

## 1. Introduction

Proprotein convertase subtilisin/kexin type 9 (PCSK9) regulates plasma low-density lipoprotein cholesterol (LDL-C) levels by binding and degrading LDL-C receptors, and thus increasing the levels of circulating LDL-C [1]. Gain-of-function mutations in the PCSK9 gene cause autosomal familial hypercholesterolemia [2,3]. In contrast, loss-of-function mutations are located in the LDL receptor (LDLR) and apolipoprotein B (APOB) genes, and are associated with decreased LDL-C levels [4]. Elevated LDL-C levels increase the risk of cardiovascular diseases [5]. Individuals with type 2 diabetes (T2D) develop dyslipidemia with elevated LDL-C levels, which are commonly treated with antihyperlipidemic drugs [6]. PCSK9 inhibitors have been demonstrated to be very effective tools for treating high circulating LDL-C levels [5]. Furthermore, PCSK9 is also expressed in tissues other than the liver, with additional effects on the immune system, glucose metabolism, pancreas and kidney [3]. Earlier studies found elevated blood PCSK9 levels in diabetics compared with non-diabetic subjects [7,8,9]. On the contrary, other investigators did not find differences in PCSK9 levels between diabetic and non-diabetic subjects [10,11].

Type 2 diabetes is characterized by a chronic hyperglycemia, due to resistance to the insulin action in the cells of the body [12]. T2D is preceded by prediabetes, in which the blood glucose levels are higher than normal, but still below the criterion for diagnosis of T2D [13]. In a recent article, circulating PCSK9 levels were positively associated with glucose homeostasis parameters, such as insulin and hemoglobin A1c (HbA1c) [6,14]. In addition, a possible role of PCSK9 inhibitors in the risk for development of T2D has also been discussed [15,16]. Colhoun et al. [15] found that the use of the PCSK9 inhibitor did not lead to the development of new-onset diabetes, while de Carvalho and colleagues [16] showed increased plasma glucose and HbA1c after the treatment with the PCSK9 inhibitor, but they did not find an increased incidence risk for diabetes. Currently, the connection between PCSK9 and impaired blood glucose homeostasis is not clear.

PCSK9 was detected by Western blotting in human aortic smooth muscle cells and carotid atherosclerotic plaques [17]. However, PCSK9 mRNA could not be detected in monocytes or macrophages. The protein is secreted by smooth muscle cells and decreases LDLR expression in macrophages, affecting foam cell formation. Short-term treatment with PCSK9 inhibitors reduced arterial stiffness in patients with familiar hypercholesterolemia, or procedures of percutaneous transluminal coronary angioplasty [18]. Circulating PCSK9 correlated positively with high sensitive C-reactive protein (CRP) in patients with stable coronary artery disease or acute coronary syndromes [19,20].

Statin treatment increased PCSK9 (neural apoptosis-regulated convertase-1; NARC-1) mRNA expression in human liver cancer cell line (HepG2) cells and primary hepatocytes [21]. In patients with high LDL levels, statin therapy significantly increased plasma fasting PCSK9 levels in subjects using atorvastatin 40 mg/day, while lower amounts (10 mg/day) did not affect serum PCSK9 levels in a limited number of study subjects [22]. In another study, however, even a 10 mg/day dose of atorvastatin was enough to significantly raise plasma PCSK9 levels [23]. In the latter study, the PCSK9 plasma values were 100-fold higher than in the previous study using an immunoblot system. In prediabetic subjects (PreDM) receiving statins, PCSK9 plasma levels were higher compared with non-medicated subjects [24], while statin-treated patients with symptoms of coronary artery disease showed no difference in their PCSK9 plasma levels, compared with non-treated patients with similar symptoms [25]. Thus, the effects of statins on blood PCSK9 levels need further investigation.

The aim of the present study was to determine plasma PCSK9 levels and metabolic profiles of elderly subjects with different glucose tolerance profiles (normal glucose tolerance (NGT), prediabetes (PreDM) and type 2 diabetes (T2D)) under statin therapy.

## 2. Materials and Methods

**Figure 1:**
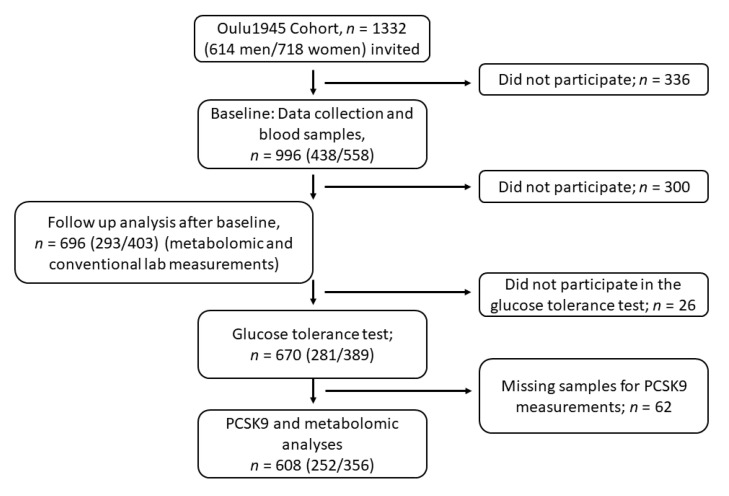
Flow chart of the study. PCSK9 (Proprotein convertase subtilisin/kexin type 9).

**Figure 2:**
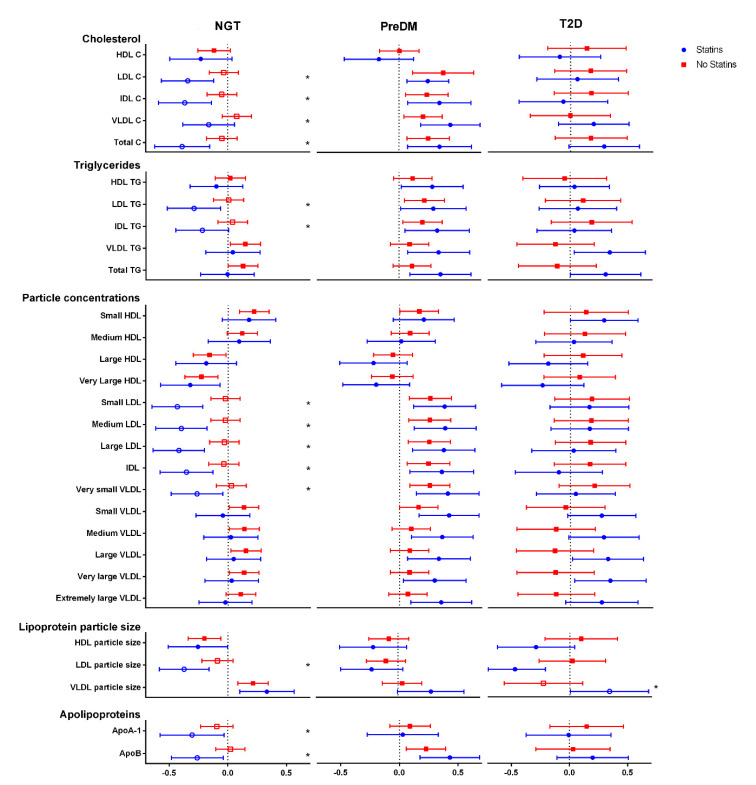
Associations between plasma NMR lipids and apolipoprotein metabolites, and Proprotein convertase subtilisin/kexin type 9 (PCSK9) in normal glucose tolerance (NGT), prediabetes (PreDM) and type 2 diabetes (T2D) subjects. *n* = 608, regression coefficients shown, * ≤ 0.05. HDL, high-density lipoprotein; LDL, low-density lipoprotein; VLDL, very low-density lipoprotein; IDL, intermediate-density lipoprotein; C, cholesterol; TG, triglyceride; ApoA-1, Apolipoprotein A1; ApoB, Apolipoprotein B.

**Figure 3:**
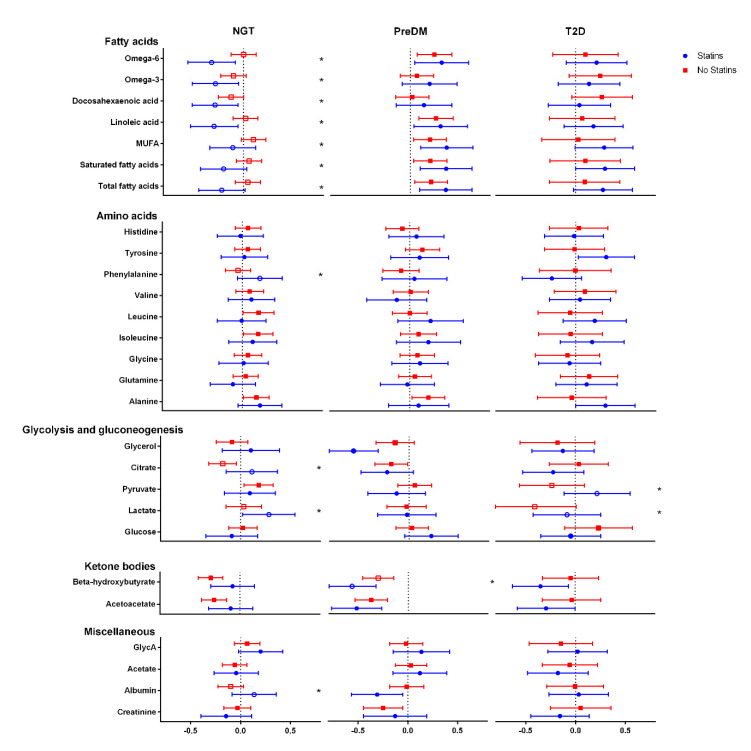
Associations between plasma NMR free fatty acids and other metabolites, and Proprotein convertase subtilisin/kexin type 9 (PCSK9) in normal glucose tolerance (NGT), prediabetes (PreDM) and type 2 diabetes (T2D) subjects. *n* = 608, regression coefficients shown, * ≤ 0.05. MUFA, Monounsaturated fatty acid.

We investigated individuals that were born in 1945 and who live in the city of Oulu (Oulu Cohort 1945), which is located in North Ostrobothnia, Finland (65° North). The original data collection was done between the years 2001–2002, and it included 1332 participants (Figure 1) [26]. In this study, we used data from the follow-up health examinations, which were carried out from 2013–2015. Blood samples were taken from 696 subjects, and oral glucose tolerance tests were done for 670 participants (281 men and 389 women) [27]. PCSK9 and metabolomics analyses were performed from 608 subjects (Figure 2 and Figure 3). The subjects continued their prescribed medication, and the use of statin medication was asked about by questionnaire. Statin medication mainly consisted of atorvastatin and simvastatin. Blood pressure (BP) was determined with an automated blood pressure monitor (Omron M3, Omron Healthcare Europe B.V., Hoofddorp, The Netherlands). Waist circumference, height and weight were measured with a standardized protocol. The habitual physical activity of the participants was measured objectively with a wrist-worn acceleration meter for two weeks (Polar Active, Polar Electro Oy, Kempele, Finland) [28].

Fasting plasma was collected between 8 and 10 a.m. in ethylenediaminetetraacetic acid (EDTA) tubes after a 12 h overnight fast, stored at −72 °C and analyzed using a high-throughput NMR metabolomics platform, which provides lipoprotein subclasses, lipids, cholesterol, amino acids, ketone bodies, glycolytic precursors and proteins [29]. Plasma levels of PCSK9 were determined using PCSK9 ELISAs (Human Proprotein Convertase 9/PCSK9, R&D SYSTEMS^TM^, Oxon, United Kingdom). The reported precisions within an assay for three test samples were between 4.1% and 6.5%, and the inter-assay precisions were between 4.1% and 5.9%. We have previously verified the antibody used in the kits as specific to PCSK9 [24].

A standardized 75 g oral glucose tolerance test (OGTT) was performed, and fasting and the 2 h glucose levels were determined from capillary blood samples. HbA1c was analyzed with the Bayer DCA2000 Analyzer (Siemens Healthcare GmbH, Erlangen, Germany) Serum immunoreactive insulin concentration was measured by radioimmunoassay (RIA) using the Phadeseph Insulin RIA100 kit (Pharmacia Diagnostics AB, Uppsala, Sweden) [30]. The fatty liver index (FLI) was calculated for each participant as a non-invasive method of assessing hepatic steatosis, according to Bedogoni et al. [31]. Alcohol consumption was evaluated by frequency, type and amount, and translated into grams of alcohol/day. Smoking habits were asked about, and subjects were categorized as current, former (quit > six months earlier) and never smokers.

Continuous variables are presented as means and standard deviations (SD), and categorical variables are presented as the number and percentage of subjects in each category. For categorical variables, Pearson’s chi-squared test or Fisher’s exact test were used to identify any differences in proportions between categorical variables. The differences in the distribution of clinical characteristics were tested using a non-parametric Mann–Whitney U test or Kruskal–Wallis test.

Multivariable linear regression was used to examine the association between PCSK9 and metabolite, and the analyses were adjusted for sex and body mass index (BMI). All metabolic measures were log-transformed to achieve normal distributions, and then mean-centered and unit-variance scaled.

In addition, the associations were estimated stratifying on statin therapy between different glucose profiles. A *p*-value of *p* < 0.05 was considered statistically significant. Statistical analyses were conducted using the free software package R 4.0.2 (https://www.r-project.org/ (accessed on 21 January 2021)).

## 3. Results

The study characteristics and clinical data are shown in Table 1. The study included elderly male and female participants with a mean age of 68.9 years. BMI and waist circumference were significantly different between the groups (*p* < 0.001), with the lowest values in the NGT group. Fasting glucose and fasting insulin were higher in PreDM and T2D groups compared with the NGT subjects, with a significant difference between the groups (*p* < 0.001). The homeostatic model assessment for insulin resistance (HOMA2-IR) and insulin sensitivity (HOMA2-S) significantly differed among the three groups, with the highest values in the T2D group. Among the lipids, total cholesterol was significantly altered between the groups (*p* < 0.001). PreDM individuals had the highest levels, while the T2D group showed the lowest circulating lipid levels. The highest high-density lipoprotein cholesterol (HDL-C) levels were found in the NGT subjects, and the groups were significantly different. Triglycerides reached the highest levels in PreDM and T2D subjects, while NGT individuals had the lowest values (*p* < 0.001 among all study groups). High-sensitivity C-reactive protein (h-CRP) levels were higher in the PreDM and T2D groups compared with the NGT group; there was a significant difference between the groups (*p* < 0.001). Other characteristics/clinical data are presented in Table 1.

LDL-C levels were significantly different (*p* < 0.001) between the three study groups, regardless of the statin medication status (Table 2; no statin: NGT (1.7 ± 0.5), PreDM (1.7 ± 0.5) and T2D (1.4 ± 0.6); statin medication: NGT (1.2 ± 0.4), PreDM (1.3 ± 0.5), T2D (1.0 ± (0.3)). In the subjects without statin medication, very low-density lipoprotein cholesterol (VLDL-C) levels were significantly different among the three groups (*p* < 0.001, NGT: 0.6 ± (0.2), PreDM: 0.7 ± (0.2), T2D: 0.7 ± (0.4)). In contrast, under statin medication, VLDL-C levels were similar among the groups (*p* = 0.298). PCSK9 plasma levels (ng/mL) had a high standard variation but did not differ between the groups regardless of the statin medication status (no statin (*p* = 0.258): NGT (251.6 ± 77.0), PreDM (248.5 ± 70.5) and T2D (275.5 ± 215.1); statin medication (*p* = 0.751): NGT (326.3 ± 136.1), PreDM (331.9 ± 74.3), T2D (315.6 ± 85.5)). PCSK9 significantly increased under statin medication in each group (Table 2). Under statins, LDL-C and VLDL-C levels were significantly decreased in subjects in each group (*p* < 0.001 or 0.001, Table 2).

Statin usage affected the association between PCSK9 and lipid metabolism variables in subjects with normal glucose tolerance with more negative associations between PCSK9 and LDL-C, intermediate-density lipoprotein cholesterol (IDL-C), VLDL-C and total cholesterol levels (NGT; Figure 2). LDL and IDL triglycerides, small LDL, medium LDL, large LDL, IDL and very small VLDL particle concentrations followed a similar trend. Similar observations were made with LDL particle size, apolipoproteins and fatty acids as well as lactate, while there were more positive associations for phenylalanine, citrate, lactate and albumin with the statin medication (Figure 3). In subjects with impaired glucose metabolism and T2D, statin usage had no significant effects on the associations between PCSK9 and metabolomics parameters, with very few exceptions (VLDL particle size, pyruvate and lactate).

## 4. Discussion

Our study illuminates the associations of statin therapy with PCSK9 and metabolic effects in subjects with elevated blood levels. Our results demonstrate that in an increased diabetic environment (PreDM, T2D), the associations between PCSK9 and lipid classes significantly change, yet the potency of statin medication was not diminished in the diabetic subjects. Plasma PCSK9 levels had a significant variation and were elevated in diabetic subjects, compared with control subjects [8]. Brouwers et al. studied PCSK9 levels in subjects with normal or impaired glucose tolerance and T2D, and found no difference in PCSK9 concentrations between the study subjects with mean levels of between 79.6 and 81.7 ng/mL [11]. Similarly, no significant differences were observed in plasma PCSK9 levels between non-diabetic subjects and T2D patients [10]. In our cross-sectional study, PCSK9 plasma levels also did not significantly differ between the subjects in NGT, PreDM and T2D groups with no statin medication, with means of 248.5 and 275.5 ng/mL. Similarly, no significant differences were observed between the groups with statin medication with means of 315.6 and 331.9 ng/mL, yet there was a significant effect by the statin medication compared to no statin treatment (*p* < 0.001).

Statins are used to lower the LDL-C levels in the blood [5]. As expected, in our study cohort, statins were associated with lower LDL-C levels in each study group, compared with the subjects that were not under statin medication. T2D subjects with statin therapy had the lowest LDL-C levels compared with the other two groups. The higher plasma PCSK9 levels in statin users in NGT, PreDM and T2D groups compared with the non-statin users are in line with our earlier results, demonstrating that prediabetic subjects under statin therapy had higher plasma PCSK9 levels compared with untreated subjects [24]. Statin treatment blocks cholesterol synthesis, triggering the activation of the sterol regulatory element-binding protein 2 (SREBP2), which results in an increased transcription of PCSK9 and LDLR turnover [1,21]. Arsenault et al. demonstrated that PCSK9 correlated dose-dependently with atorvastatin [32]. Interestingly, in a recent analysis of 539 subjects with coronary artery disease (60.3 ± 8.6 years, 256 males), statin treatment did not significantly affect PCSK9 plasma levels (222 ± 1 and 202 ± 9 ng/mL), and the significant correlation between LDL-C and PCSK9 was lost in the statin-treated subjects, compared with non-statin users [25].

PCSK9 inhibitors have been evaluated for the treatment of high blood LDL-C as a better alternative for statins [5]. The use of PCSK9 inhibitors lowers the plasma levels of several lipids, but not VLDL-C [33,34,35]. PCSK9 plasma levels were positively correlated with VLDL-C levels in 52 middle-aged subjects, but the correlation was lost in the multivariable linear regression analyses [36]. Sullivan et al. found no correlation between PCSK9 and VLDL triglycerides in obese, non-diabetic subjects [37]. In our study, VLDL-C levels were significantly different between the study groups with no lipid medication, but not in the subjects with impaired glucose tolerance. Thus, our results support the earlier findings, suggesting that VLDL-C metabolism is not affected by PSCK9 in subjects with abnormal glucose metabolism.

Several blood lipids like LDL-C, IDL-C, VLDL-C, total cholesterol levels, LDL and IDL triglycerides, small LDL, medium LDL, large LDL, IDL and very small VLDL particle concentrations were associated with PCSK9 levels in NGT subjects using statins compared with the non-statin users. However, in prediabetic and diabetic individuals, these associations were lost. Insulin by itself stimulates the mechanistic target of rapamycin complex 1 (mTORC1) kinase, a master controller of metabolism, which inhibits the transcription factor hepatocyte nuclear factor (HNF4α and HNF1α), and thus transcription of PCSK9 [38]. PCSK9 knock-out mice show impaired insulin secretion when compared with wild-type control mice [39]. In humans, the PCSK9 variant 46 L has been connected to pancreatic beta-cell dysfunction [40]. The use of statins has been linked to increased risk of T2D [41], but PCSK9 inhibitors have also been implicated in the development of diabetes [15,16] and further long-term observations are required.

Our study has several strengths and weaknesses. Our investigation is limited by the cross-sectional analysis in Northern Finland. In addition, we needed to rely on self-reported medication, and we did not have information about the length of the statin treatment or possible apolipoprotein E (APOE) polymorphisms. In the Dallas Heart Study, the prevalence of the APOE polymorphism was 0.51% [9], which would translate into three subjects in our 608 measured subjects, and would therefore not significantly affect our results. The strengths of our study are that our subjects are almost the same age, and have similar environment and ethnicity. We performed an oral glucose tolerance test in all subjects not previously diagnosed with T2D, and physical activity levels were objectively recorded by an activity meter over a 2-week period.

## 5. Conclusions

In the present study, we analyzed the plasma PCSK9 levels and metabolic profiles of elderly NGT, PreDM and T2D subjects with or without statin therapy. Our data demonstrate that under disturbed glucose homeostasis (PreDM, T2D), PCSK9 levels were further significantly increased by statin therapy. Due to these increases with corresponding changes in lipid metabolism under statin therapy, the associations (e.g., LDL-C, IDL-C, VLDL-C total cholesterol levels) of the PCSK9-mediated changes were lost. In the abnormal glucose homeostasis, the clinical effects of statins were maintained, but further studies would need to evaluate if PCSK9 inhibitors might be clinically more beneficial, and also in terms of additional systemic effects (e.g., glucose homeostasis, pancreas, kidney, inflammation).

## Figures and Tables

**Table 1:** Anthropological and biochemical measurements. Means and standard deviations in brackets or numbers and percent of subjects (*n*; %) are shown.

	Total	NGT	PreDM	DM	*p*-Value
	*n* = 670	*n* = 348	*n* = 217	*n* = 105	
**Age**	68.9 (0.55)	68.9 (0.57)	68.9 (0.52)	68.8 (0.55)	0.239
**Sex**					**0.002**
Men	281 (41.9%)	132 (37.9%)	89 (41.0%)	60 (57.1%)	
Women	389 (58.1%)	216 (62.1%)	128 (59.0%)	45 (42.9%)	
**BMI (kg/m^2^)**	27.6 (4.69)	26.0 (3.75)	28.6 (4.70)	30.7 (5.31)	**<0.001**
Normal	201 (30.3%)	140 (40.8%)	49 (22.6%)	12 (11.5%)	
Overweight	293 (44.1%)	162 (47.2%)	94 (43.3%)	37 (35.6%)	
Obese	170 (25.6%)	41 (12.0%)	74 (34.1%)	55 (52.9%)	
**Waist circumference (cm)**	94.0 (13.7)	88.9 (11.5)	97.0 (12.8)	105 (14.0)	**<0.001**
**Fasting glucose (mmol/L)**	5.75 (1.03)	5.31 (0.41)	5.88 (0.55)	7.02 (1.85)	**<0.001**
**Fasting insulin (pmol/L)**	15.4 (17.0)	11.3 (5.62)	16.1 (9.42)	27.8 (37.3)	**<0.001**
**HOMA2-B**	109 (42.0)	108 (33.7)	112 (42.9)	106 (62.1)	0.406
**HOMA2-S**	71.7 (39.9)	82.4 (38.7)	63.2 (38.4)	52.3 (35.8)	**<0.001**
**HOMA2-IR**	1.91 (1.36)	1.48 (0.71)	2.12 (1.20)	2.98 (2.42)	**<0.001**
**DP (mmHg)**	85.6 (9.73)	84.1 (9.50)	88.1 (9.44)	85.2 (10.1)	**<0.001**
**SP (mmHg)**	144 (17.7)	141 (17.2)	148 (18.0)	147 (16.6)	**<0.001**
**Daily steps**	8869 (3696)	9283 (3613)	8499 (3686)	8118 (3885)	**0.024**
**Physical activity questionaires**					**<0.001**
Non-active ^a^	153 (23.3%)	62 (18.1%)	46 (21.4%)	45 (44.6%)	
Active ^b^	505 (76.7%)	280 (81.9%)	169 (78.6%)	56 (55.4%)	
**Smoking:**					**0.001**
Current	81 (12.4%)	39 (11.4%)	20 (9.5%)	22 (21.8%)	
Former (>6 months earlier)	225 (34.4%)	107 (31.2%)	75 (35.5%)	43 (42.6%)	
Never	349 (53.2%)	197 (57.4%)	116 (55.0%)	36 (35.6%)	
**Alcohol (g/d)**	1.85 (4.61)	1.23 (2.29)	2.80 (7.11)	1.99 (3.37)	**<0.001**
**Total cholesterol (mmol/L)**	5.33 (1.22)	5.44 (1.15)	5.51 (1.18)	4.60 (1.26)	**<0.001**
**HDL-C (mmol/L)**	1.65 (0.46)	1.74 (0.46)	1.61 (0.43)	1.40 (0.38)	**<0.001**
**Triglycerides (mmol/L)**	1.27 (0.81)	1.07 (0.42)	1.42 (0.70)	1.63 (1.53)	**<0.001**
**HbA1c (mmol/L)**	40.6 (5.87)	38.9 (3.45)	40.2 (4.00)	47.1 (9.95)	**<0.001**
**FLI**	45.6 (28.9)	33.2 (23.9)	54.5 (28.0)	68.0 (25.9)	**<0.001**
**h-CRP (mg/L)**	3.49 (9.47)	2.30 (4.42)	4.49 (12.7)	5.36 (13.0)	**0.002**

BMI: body mass index; HOMA2-B, HOMA2-S and HOMA2-IR: homeostatic model assessment for beta-cell function, insulin sensitivity, and insulin resistance, respectively; DP: diastolic pressure; SP: systolic pressure; ^a^: <30 min light exercise, e.g., walking/week; ^b^: >30 min light exercise, e.g., walking/at least once a week; HDL-C: high-density lipoprotein cholesterol; HbA1c: hemoglobin A1c; FLI: fatty liver index; h-CRP: high-sensitivity C-reactive protein; GFR: glomerular filtration rate. *p*-values < 0.05 are considered significant (in bold format).

**Table 2:** The effect of statin medication of circulating PCSK9 (based on ELISA measurements), LDL-C and VLDL-C levels (based on NMR metabolomics measurements) in NGT, PreDM and T2D subjects.

	Analyte	No Statin (*n* = 427)	Statin Medication (*n* = 181)	Total (*n* = 608)	*p*-Value
**PCSK9 (ng/mL)**	**NGT**	251.6 (77.0)	326.3 (136.1)	270.6 (100.7)	**<0.001**
	**PreDM**	248.5 (70.5)	331.9 (74.3)	271.4 (80.6)	**<0.001**
	**T2D**	275.5 (215.1)	315.6 (85.5)	295.3 (164.7)	**<0.001**
	***p*** **-value**	0.258	0.751	0.131	
**LDL-C (mmol/L)**	**NGT**	1.7 (0.5)	1.2 (0.4)	1.6 (0.5)	**<0.001**
	**PreDM**	1.7 (0.5)	1.3 (0.5)	1.6 (0.5)	**<0.001**
	**T2D**	1.4 (0.6)	1.0 (0.3)	1.2 (0.5)	**<0.001**
	***p*** **-value**	**<0.001**	**<0.001**	**<0.001**	
**VLDL-C (mmol/L)**	**NGT**	0.6 (0.2)	0.5 (0.2)	0.6 (0.2)	**<0.001**
	**PreDM**	0.7 (0.2)	0.5 (0.2)	0.7 (0.2)	**<0.001**
	**T2D**	0.7 (0.4)	0.5 (0.2)	0.6 (0.3)	**0.001**
	***p*** **-value**	**<0.001**	0.298	**<0.001**	

Means ± standard deviations are shown in the table. *p*-values < 0.05 are considered significant (in bold format).

## Data Availability

Data available on request due to restrictions (e.g., privacy or ethical).
